# Pressure‐Driven Reactivity in Dense Methane‐Nitrogen Mixtures

**DOI:** 10.1002/anie.202422710

**Published:** 2025-04-03

**Authors:** Hannah A. Shuttleworth, Mikhail A. Kuzovnikov, Lewis J. Conway, Huixin Hu, Jinwei Yan, Samuel Gallego‐Parra, Israel Osmond, Tomas Marqueño, Michael Hanfland, Dominique Laniel, Eugene Gregoryanz, Andreas Hermann, Miriam Peña‐Alvarez, Ross T. Howie

**Affiliations:** ^1^ Centre for Science at Extreme Conditions University of Edinburgh Edinburgh EH9 3FD United Kingdom; ^2^ Center for High Pressure Science and Technology Advanced Research 1690 Cailun Road Shanghai 201203 China; ^3^ Department of Materials Science & Metallurgy University of Cambridge 27 Charles Babbage Road Cambridge CB3 0FS United Kingdom; ^4^ European Synchrotron Radiation Facility 71 Avenue des Martyrs 38000 Grenoble France; ^5^ Key Laboratory of Materials Physics Institute of Solid State Physics, CAS Hefei 230031 China; ^6^ SHARPS (Shanghai Advanced Research in Physical Sciences) 68 Huatuo Road Shanghai 201203 China

**Keywords:** high-pressure chemistry, raman spectroscopy, synchrotron x-ray diffraction, molecular systems, high-temperature chemistry

## Abstract

Carbon, nitrogen, and hydrogen are among the most abundant elements in the solar system, and our understanding of their interactions is fundamental to prebiotic chemistry. CH_4_ and N_2_ are the simplest archetypical molecules formed by these elements and are both markedly stable under extremes of pressure. Through a series of diamond anvil cell experiments supported by density functional theory calculations, we observe diverse compound formation and reactivity in the CH_4_‐N_2_ binary system at high pressure. Above 7 GPa two concentration‐dependent molecular compounds emerge, (CH_4_)_5_N_2_ and (CH_4_)_7_(N_2_)_8_, held together by weak van der Waals interactions. Strikingly, further compression at room temperature irreversibly breaks the N_2_ triple bond, inducing the dissociation of CH_4_ above 140 GPa, with the near‐quenched samples revealing distinct spectroscopic signatures of strong covalently bonded C−N−H networks. High temperatures vastly reduce the required pressure to promote the reactivity between CH_4_ and N_2_, with NH_3_ forming together with longer‐chain hydrocarbons at 14 GPa and 670 K, further decomposing into powdered diamond when temperatures exceed 1200 K. These results exemplify how pressure‐driven chemistry can cause unexpected complexity in the most simple molecular precursors.

## Graphical abstract

CH_4_ and N_2_ are abundant molecules in our solar system and are the primary constituents of Titan's atmosphere. Under extreme pressures and temperatures within a diamond anvil cell, CH_4_ and N_2_ are demonstrated to react to form a range of compounds, dependent on the conditions and initial concentrations. This provides valuable insight into the complexity of the C−N−H ternary system under planetary conditions.

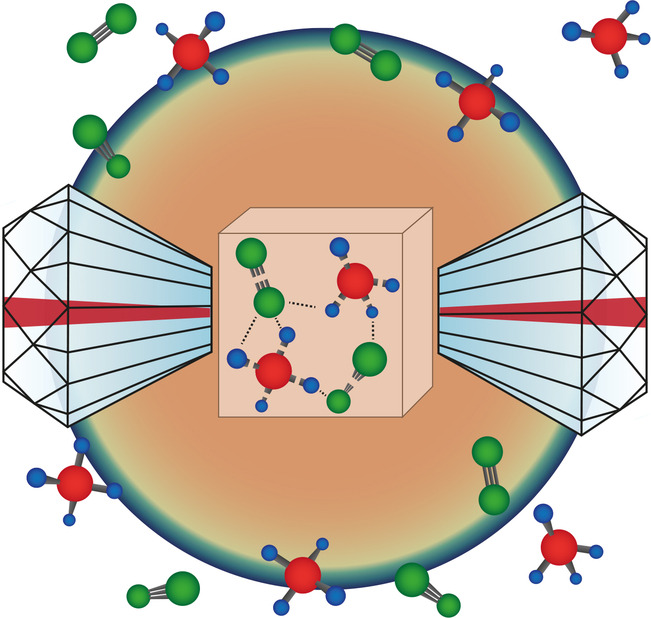

## Introduction

Carbon, nitrogen, and hydrogen constitute some of the most prevalent elements in the solar system, and exploring their chemical interactions is crucial to understanding the origin of life.^[1]^ To explore the chemical interplay between these elements at planetary relevant conditions, the effect of pressure must be considered in addition to temperature‐induced chemistry. By altering molecular orbitals and interactions, high pressure serves as a clean chemical method for the synthesis of novel materials that would otherwise be unstable at ambient pressure.[^2^, ^3^, ^4^, ^5^, ^6^, ^7^, ^8^, ^9^, ^10^, ^11^, ^12^] A key example of this is how the stability and reactivity of molecular materials evolve upon compression. Due to the extremely strong N≡N triple bond, molecular N_2_ is one of the least reactive molecules under ambient conditions. However, high pressure has been shown to induce the formation of numerous non‐molecular phases of nitrogen, requiring the breaking of the triple bond.[^13^, ^14^, ^15^, ^16^, ^17^, ^18^, ^19^, ^20^, ^21^, ^22^] The potential chemistry of N_2_ at high pressure is further enhanced with temperature, resulting in the synthesis of novel polynitrides, which are of key interest due to their potential as high energy density compounds.[^4^, ^10^, ^23^, ^24^, ^25^, ^26^, ^27^, ^28^]

Like nitrogen, methane is another example of a simple molecular system that undergoes a series of physical and structural changes under pressure, with crucial implications in understanding physical processes within planetary interiors.[^29^, ^30^, ^31^] Both theoretical and experimental studies have demonstrated that the decomposition of methane into longer hydrocarbons and diamond is favored when compressed and heated.[^29^, ^32^, ^33^, ^34^, ^35^, ^36^, ^37^, ^38^] Additionally, it is theorized that at room temperature and pressures approaching 300 GPa, methane will dissociate into diamond and H_2_, which is of great interest regarding the mantle region of planets such as Neptune and Uranus, where methane is abundant.[^33^, ^39^, ^40^, ^41^, ^42^]

There is observational evidence that reactions occur between CH_4_ and N_2_ in the atmosphere of the satellite Titan, whereby hydrocarbon‐nitrile aerosol compounds, named “tholins”, have formed from CH_4_‐N_2_ mixtures at low pressures and temperatures in the presence of solar ultraviolet radiation.[^43^, ^44^, ^45^, ^46^, ^47^] Recent theoretical works also predict that under dense planetary conditions, exotic organic chemistry occurs within the C−N−H system.[^48^, ^49^] A variety of stable C−N−H compounds are predicted to emerge at pressures up to 50 GPa.^[48]^ Most notably, two high energy density compounds were predicted: CN_2_H_4_ and CH_4_N_4_, stable at 11 GPa and 41 GPa respectively, the latter of which is predicted to be stable to ambient conditions upon quenching. With a calculated energy release of 6.43 KJg^−1^ upon decomposition back into molecular N_2_ and CH_4_, this is a promising high energy density compound.^[48]^

At ambient pressure, experiments have shown that CH_4_ and N_2_ can react, though require combinations of high‐temperature, electric discharge, microwave radiation, and catalysts.[^50^, ^51^, ^52^, ^53^] Most experimental approaches investigating the C−N−H system at high pressure have used complex precursors rather than the simplest archetypical molecules originating from the ternary system: CH_4_ and N_2_.[^54^, ^55^, ^56^, ^57^] There has been only one experimental study on CH_4_‐N_2_ mixtures up to 16 GPa, presenting evidence of compound formation retaining N_2_ and CH_4_ molecular units; however, the structural characterization was unsuccessful.^[58]^ Surprisingly, whether the combination of high pressure and temperature can facilitate a reaction between CH_4_ and N_2_ has yet to be explored experimentally.

In this work, we investigate pressure and temperature‐induced chemistry in the dense CH_4_‐N_2_ system. We report the formation of two CH_4_‐N_2_ van der Waals compounds above 7 GPa at room temperature from binary CH_4_‐N_2_ fluid mixtures. Through a combination of X‐ray diffraction and first principle calculations, these are identified as *P*4_2_/*mnm*‐(CH_4_)_7_(N_2_)_8_ and Ibam ‐(CH_4_)_5_N_2_. Remarkably, upon room temperature compression of either compound, we observe the irreversible pressure‐induced dissociation and the reaction of N_2_ and CH_4_ molecules above 140 GPa. Decompression of the reaction products to near‐ambient conditions reveals spectroscopic signatures of strong covalently bonded C−N−H networks. The application of high temperature can induce a reaction at substantially lower pressures, where we observe the formation of NH_3_, together with longer‐chain hydrocarbons at 670 K (at 14 GPa), the latter of which decomposes into powdered diamond when temperatures exceed 1200 K at 25 GPa.

## Results and Discussion

### Synthesis, Characterization and Stability of CH_4_‐N_2_ Molecular Compounds

**Figure 1:**
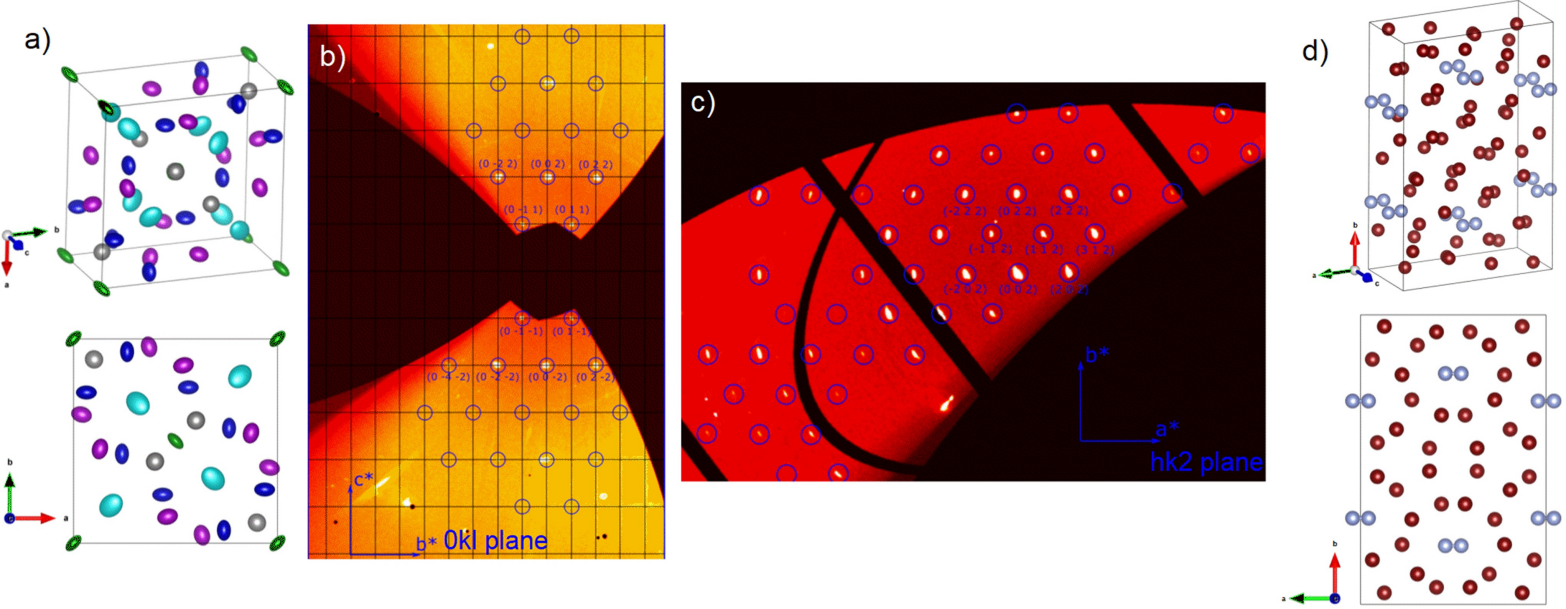
(a) Structural models of the unit cell of *P*4_2_/*mnm*‐(CH_4_)_7_(N_2_)_8_ (top) and a projection of the *P*4_2_/*mnm*‐(CH_4_)_7_(N_2_)_8_ structure along the *c*‐axis (bottom). Different colors represent different molecular sites rather than different molecular types. The shape of the ellipsoids represents the preferred orientation of the molecules. (b) Slice of the (0kl) reciprocal space of an *P*4_2_/*mnm*‐(CH_4_)_7_(N_2_)_8_ single crystal at 7 GPa. The blue circles indicate visible reflections. The systematic absences are consistent with those of space group *P*4_2_/*mnm*. (c) Reconstruction of the (hk2) reciprocal space slice built using single‐crystal data collected on Ibam ‐(CH_4_)_5_N_2_ single crystal at 13 GPa. The blue circles highlight visible reflections. The systematic absences are consistent with space group *Ibam*. (d) The unit cell of Ibam ‐(CH_4_)_5_N_2_ (top) and a projection of the Ibam ‐(CH_4_)_5_N_2_ unit cell along the *c*‐axis (bottom), nitrogen atoms are represented as blue spheres and CH_4_ molecules are represented by brown spheres.

**Figure 2:**
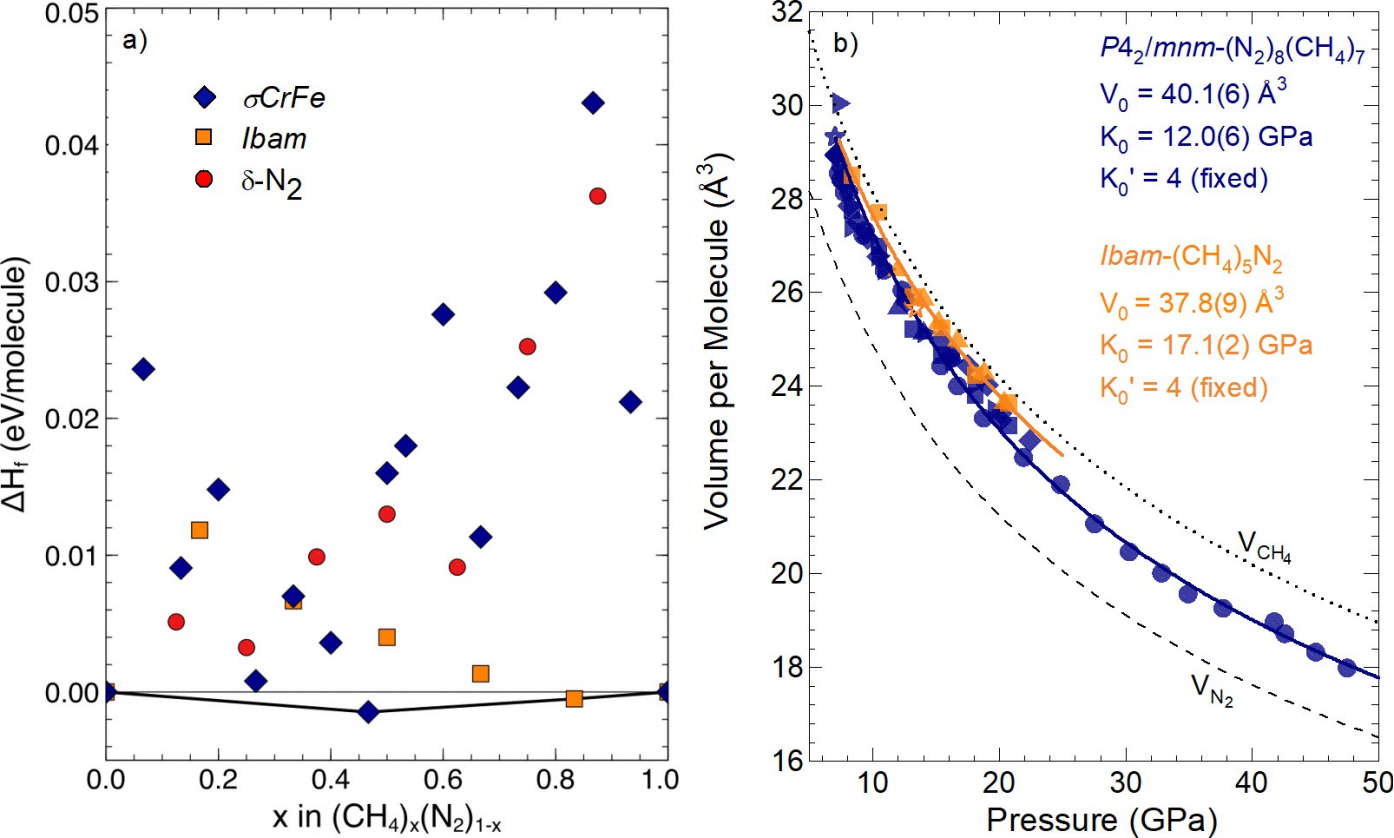
(a) Ground state relative enthalpies of CH_4_‐N_2_ mixtures from DFT calculations at 15 GPa, based on the *σ*‐CrFe, Ibam , and *δ*‐N_2_ structure types (see text). The black line indicates the convex hull of stable phases. (b) Volume per molecule of the two van der Waals compounds as a function of pressure, obtained from powder XRD data. Symbols represent experimental data for *P*4_2_/*mnm*‐(CH_4_)_7_(N_2_)_8_ (blue) and Ibam ‐(CH_4_)_5_N_2_ (orange), with stars representing SCXRD data and the remaining symbols representing powder XRD data. The solid lines represent their best Birch‐Murnaghan fit. The dashed line represents the equation of state of pure N_2_ ^[65]^ and the dotted line represents that of pure CH_4_.^[66]^

Two representative CH_4_‐N_2_ gas mixtures (50 : 50 CH_4_ : N_2_ and 67 : 33 CH_4_ : N_2_) were loaded into diamond anvil cells at 0.2 GPa (see Supporting Information for a complete description of the experimental procedure). Upon compression above 2 GPa, the fluid mixtures solidified into A‐CH_4_+*δ*‐N_2_.[^59^, ^60^, ^61^, ^62^, ^63^] Compression of the 50 : 50 mixture above 7 GPa resulted in the formation of a new compound. Single crystal X‐ray diffraction (SCXRD) data were obtained. These datasets were successfully refined to a crystal structure isomorphic to *σ*‐CrFe (space group *P*4_2_/*mnm*, Figure 1a), with lattice parameters *a*=11.904(3) Å and *c*=6.2072(13) Å, at 7 GPa (see Figure 1b and Supporting Table S1 for crystallographic data).^[64]^ The unit cell of this compound is shown in Figure 1a. Due to strong molecular rotational disorder providing limited reflections at low *d*‐spacings, along with a comparable scattering factor for N_2_ and CH_4_, we were unable to estimate site occupancies. We observed no further structural changes in our powder XRD measurements (Supporting Figure S1a) up to at least 47 GPa (Figure 2b).

Upon compression of the 67 : 33 CH_4_:N_2_ mixture to 9 GPa, we observe the presence of another uncharacterized compound. SCXRD data obtained at 13 GPa were refined to an *Ibam* unit cell (Figure 1d) with lattice parameters *a*=11.854(12) Å, *b*=18.459(5) Å and *c*=5.6278(12) Å at 13 GPa (see Figure 1c and Supporting Table S2 for crystallographic data).^[68]^ To the best of our knowledge, this is the first observation of this structural type. Out of the six distinct crystallographic positions, nitrogen could unambiguously be assigned to the 16*k* site (see Supporting Information for further details). With the five remaining sites assigned as CH_4_ molecules, the experimental data suggests a (CH_4_)_5_N_2_ composition. Extracted volumetric data from powder XRD measurements (Supporting Figure S1b) upon compression demonstrates that this compound is stable to at least 23 GPa (Figure 2b).

To ascertain the stability of the molecular compounds and estimate their compositions, we performed density functional theory (DFT) calculations (see Supporting Information for computational details). The resulting lowest formation enthalpies form part of Figure 2a, where they are shown relative to pure ϵ ‐N_2_ and pure CH_4_ (in the HP phase, *R*3‐CH_4_).^[66]^ The convex hull also includes additional data points based on the *δ*‐N_2_ structure type, which was proposed in earlier work^[58]^ to allow for some uptake of CH_4_. The only compositions that emerge as stable phases on the convex hull are *P*4_2_/*mnm*‐(CH_4_)_7_(N_2_)_8_ and Ibam ‐(CH_4_)_5_N_2_, the latter agreeing with experimental results. The Supporting Information contains molecular dynamics (MD) simulation results (which confirm the compounds’ kinetic stability and molecular rotational/librational character), composition‐volume data, and results from alternative exchange‐correlation functionals.

We have investigated both the *P*4_2_/*mnm*‐(CH_4_)_7_(N_2_)_8_ and Ibam ‐(CH_4_)_5_N_2_ phases using Raman spectroscopy, which allows us to directly explore any changes in the inter/intra‐molecular environments. At 8.5 GPa, the Raman spectrum of *P*4_2_/*mnm*‐(CH_4_)_7_(N_2_)_8_ exhibits the characteristic Raman modes of molecular CH_4_ and N_2_: the C−H bending mode $\nu_{2}$ at 1536 cm^−1^; C−H symmetric ($\nu_{1}$ ) and antisymmetric ($\nu_{3}$ ) modes at 2996 cm^−1^ and 3115 cm^−1^, respectively; and the N_2_ intramolecular vibrational modes, $\nu_{2}$ at 2341 cm^−1^ and $\nu_{1}$ at 2346 cm^−1^. At lower pressures, the Raman spectra of Ibam ‐(CH_4_)_5_N_2_ closely resembles that of *P*4_2_/*mnm*‐(CH_4_)_7_(N_2_)_8_. Upon compression, it becomes evident that the Raman frequency of the N_2_ vibrons and the CH_4_ stretching modes of each compound have a different pressure dependency, indicative that the molecular environments differ. For example, at 28 GPa, the Raman frequency of the N_2_‐$\nu_{2}$ vibron is 2372 cm^−1^ for *P*4_2_/*mnm*‐(CH_4_)_7_(N_2_)_8_, and 2367 cm^−1^ for Ibam ‐(CH_4_)_5_N_2_.

**Figure 3:**
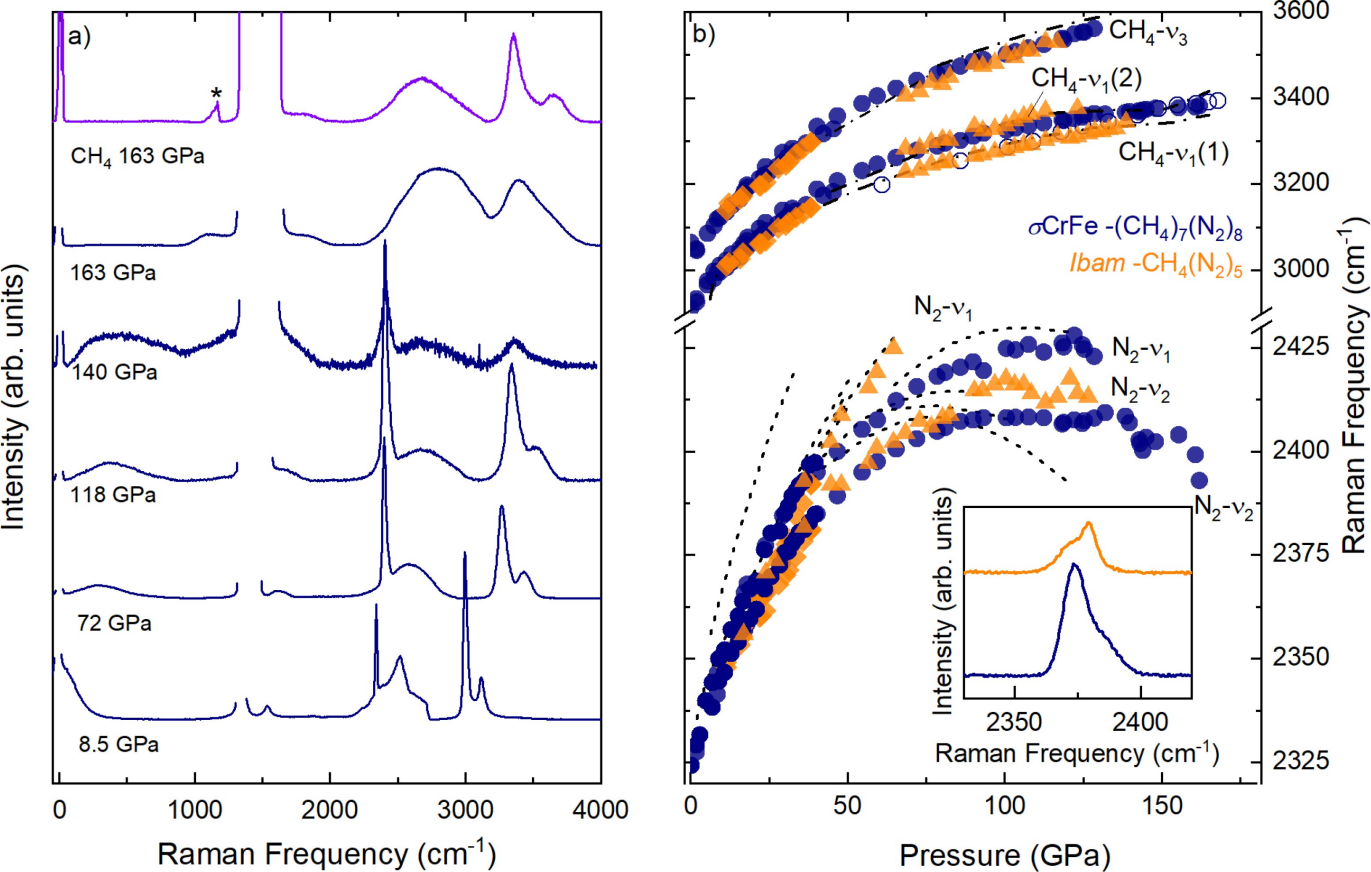
(a) Raman spectra upon compression of *P*4_2_/*mnm*‐(CH_4_)_7_(N_2_)_8_, synthesized from a 50 % N_2_ mixture, from 8.5 to 163 GPa (blue), and the Raman spectrum of pure CH_4_ at 163 GPa (lilac). The asterisk denotes a peak which is an artifact of the diamond anvils. (b) Raman shift of the N_2_ vibrational modes and C−H stretching modes of *P*4_2_/*mnm*‐(CH_4_)_7_(N_2_)_8_ (blue) and Ibam ‐(CH_4_)_5_N_2_ (orange) as a function of pressure, synthesized from a 50 % and 67 % CH_4_ gas mixture, respectively. Different symbols represent different experimental runs, and the empty symbols denote data collected upon decompression. Dotted lines represent the N_2_ vibrational modes of pure N_2_ ^[67]^ and dashed lines represent the C−H stretching modes of pure CH_4_.^[31]^ Inset: Profile of the N_2_ vibrational modes of *P*4_2_/*mnm*‐(CH_4_)_7_(N_2_)_8_ (blue) and Ibam ‐(CH_4_)_5_N_2_ (orange) at 30 GPa.

Additionally, both compounds exhibit a distinct deviation in the frequencies of the Raman active modes compared with the pure species (Figure 3a).[^31^, ^67^] For example, in pure nitrogen, there is complex splitting of the N_2_‐$\nu_{2}$ vibron at 24 GPa^[67]^ (Supporting Figure S2), whilst *P*4_2_/*mnm*‐(CH_4_)_7_(N_2_)_8_ and Ibam ‐(CH_4_)_5_N_2_ show two distinct modes. Splitting of the CH_4_‐$\nu_{1}$ into CH_4_‐$\nu_{1}$ (1) and CH_4_‐$\nu_{1}$ (2) is observed during compression of Ibam ‐(CH_4_)_5_N_2_ beyond 67 GPa^[31]^ (Figure 3b). The splitting is not resolvable in *P*4_2_/*mnm*‐(CH_4_)_7_(N_2_)_8_ due to the low intensity of the C−H stretching modes. Furthermore, inspection of the N_2_ vibrational modes reveals that the relative intensity of the N_2_‐$\nu_{1}$ and N_2_‐$\nu_{2}$ modes also depends on the compound formed, as seen in Figure 3b. In both compounds, we also see an intense broad band at frequencies close to the Rayleigh line (Figure 3a). This could be attributed to rotational disordered molecules, which is in agreement with our X‐ray diffraction measurements and MD simulations demonstrating freely rotating CH_4_ units (Supporting Figure S6).

### Pressure Induced Reactivity in the CH_4_‐N_2_ System

While CH_4_ is stable to at least 200 GPa,[^31^, ^69^] the triple bond of molecular N_2_ undergoes progressive weakening to the extent that polymeric allotropes form above 110 GPa.[^13^, ^14^, ^15^, ^16^, ^17^, ^18^, ^19^, ^20^, ^21^, ^22^] We investigated the reactivity of *P*4_2_/*mnm*‐(CH_4_)_7_(N_2_)_8_ (Figure 3a) and Ibam ‐(CH_4_)_5_N_2_ (Supporting Figure S3) phases using Raman spectroscopy. Compression of *P*4_2_/*mnm*‐(CH_4_)_7_(N_2_)_8_ led to a turnover in the frequencies of the N_2_ vibrons above 100 GPa and subsequent softening of the modes (Figure 3b). At 128 GPa, the higher frequency $\nu_{1}$ vibron becomes unidentifiable, while the N_2_‐$\nu_{2}$ mode is unresolvable above 163 GPa in *P*4_2_/*mnm*‐(CH_4_)_7_(N_2_)_8_ and 136 GPa in Ibam ‐(CH_4_)_5_N_2_. Before disappearing, the frequency of the N_2_ vibrational mode in *P*4_2_/*mnm*‐(CH_4_)_7_(N_2_)_8_ at 163 GPa is comparative to the frequency observed in pure N_2_ at the onset of polymerization (120 GPa).[^16^, ^19^] This is indicative of pressure‐induced breaking of the N_2_ triple bond to form polymeric nitrogen.[^13^, ^14^, ^16^, ^67^, ^70^, ^71^, ^72^, ^73^] Concurrently, a new mode at around 1200 cm^−1^ appears, corresponding to vibrational modes of single bonded C−N and C−C.[^74^, ^75^, ^76^, ^77^] Additionally, the CH_4_‐$\nu_{2}$ bending mode, along with the symmetric ($\nu_{1}$ ) and anti‐symmetric ($\nu_{3}$ ) stretching modes, decreases in intensity and broadens (Figure 3a). A direct comparison to the Raman spectrum of CH_4_,^[31]^ which we measured at 163 GPa to provide a reference (Figure 3a), indicates dissociation and formation of a reaction product. No detectable X‐ray diffraction patterns were obtained at this pressure, likely due to weak scattering or sample amorphization, similar to η ‐nitrogen.^[14]^

**Figure 4:**
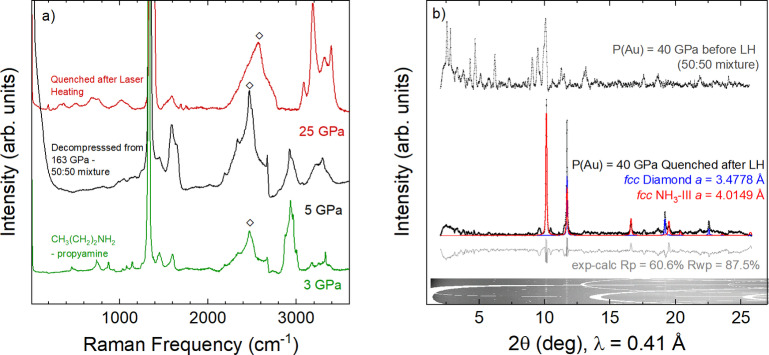
(a) Raman spectrum of NH_3_, powdered diamond and hydrocarbons at 25 GPa, formed after laser heating *P*4_2_/*mnm*‐(CH_4_)_7_(N_2_)_8_ (red), the Raman spectrum after compression of a 50 % CH_4_ mixture to 163 GPa then decompression to 5 GPa (black), and the Raman spectrum of propylamine (CH_3_(CH_2_)_2_(NH)_2_) at 3 GPa (green). The diamond symbols represent the second‐order diamond Raman mode. (b) Rietveld refinement of *fcc* NH_3_‐III and powdered diamond quenched to ambient temperature, formed after laser heating *P*4_2_/*mnm*‐(CH_4_)_7_(N_2_)_8_ to above 1200 K at 40 GPa. The diffraction pattern of *P*4_2_/*mnm*‐(CH_4_)_7_(N_2_)_8_ before laser heating is also shown.

Strikingly, decompression of the reaction products to 5(4) GPa, does not result in the transformation back into either molecular compound nor do the Raman signatures of molecular N_2_ reappear (Figure 4a and Supporting Figure S3), unlike pure N_2_ whereby the vibrons re‐emerge at 40 GPa.^[67]^ Similarly, the C−H stretching modes remain broad compared to either molecular compound at comparative pressures, while the band between 1100–1200 cm^−1^ remains active but as a broad series of peaks, which we attribute to the formation of oligomers.^[74]^ These Raman features are observed regardless of whether the reaction products of *P*4_2_/*mnm*‐(CH_4_)_7_(N_2_)_8_ or *Ibam*‐(CH_4_)_5_N_2_ are decompressed.

By comparing the experimental data with the known Raman spectra of the simplest covalent C−H−N compounds, amines, we tentatively assign it to propylamine (CH_3_(CH_2_)_2_NH_2_). As such, we measured the Raman spectra of propylamine up to 3 GPa to provide a direct comparison (Figure 4). Here, we see reasonable agreement between the modes around 3100–3450 cm^−1^, attributed to N−H stretching, together with C−H stretching modes in the 2800–3000 cm^−1^ region.^[76]^ Deformation modes within the 1400–1500 cm^−1^ region and C−H bending in the 1560–1580 cm^−1^ region are in good agreement with those of our products.[^76^, ^77^, ^78^] We also see an overlap in the N−H stretching region, indicating the presence of additional molecular units. Unfortunately, attempts at structural characterization of the reaction products were unsuccessful, which we attribute to the small sample sizes combined with the weak X‐ray scattering of the constituent elements, whilst full decompression led to the loss of the samples. In recent theoretical works exploring compound formation in the C−N−H system, a molecular compound composed of propylamine, NH_3_, and CH_4_ units is predicted to be the most thermodynamically stable reaction product in CH_4_‐N_2_ mixtures below 10 GPa.^[48]^ As such, we propose this molecular compound, CH_3_(CH_2_)_2_NH_2_+NH_3_+CH_4_, as the most promising candidate for the compression product. Interestingly, amines, including propylamine, are believed to be a constituent of Titan's “tholins”.[^79^, ^80^]

Given that temperature is known to destabilize both CH_4_ and N_2_ at high pressure, we explored the high temperature stability of the molecular compounds in an attempt to synthesize covalently bonded C−N−H compounds at lower pressures.^[48]^ Upon heating Ibam ‐(CH_4_)_5_N_2_ phase to temperatures above 670 K at 14 GPa, we observe a reaction to form NH_3_ as evidenced by our X‐ray diffraction measurements (see Supporting Figure S4).^[81]^ Laser heating either *P*4_2_/*mnm*‐(CH_4_)_7_(N_2_)_8_ or Ibam ‐(CH_4_)_5_N_2_ precursors at 40 GPa to temperatures in excess of 1200 K also led to a reaction, with the temperature quenched diffraction pattern showing the formation of NH_3_‐III together with *fcc* diamond (*a*=3.4778 Å) (Figure 4b and Supporting Figure S5). The presence of powdered diamond is a result of the decomposition of CH_4_ into C, with H_2_ reacting to form NH_3_ and longer chain hydrocarbons when laser heated.[^29^, ^33^, ^34^] Raman spectroscopy experiments of the quenched sample support these observations, with the N_2_ vibrons becoming unresolvable and the NH_3_ stretching modes, *ν*_1_, 2*ν*_4_ and *ν*_3_, appearing at 3187 cm^−1^, 3318 cm^−1^ and 3395 cm^−1^ at 25 GPa. The C−H stretching mode *ν*_1_, additional modes in the C−H bending region 1460–1800 cm^−1^, and a complex spectrum below 1330 cm^−1^ suggest the presence of a mixture of longer‐chain light hydrocarbons.[^36^, ^82^] Such mixtures have been previously established as oligomers, which are undetectable in X‐ray diffraction experiments.^[83]^

## Conclusion

By compressing mixtures of the simple CH_4_‐N_2_ binary molecular system in a series of diamond anvil cell experiments, we report the formation of two molecular van der Waals compounds, Ibam ‐(CH_4_)_5_N_2_ and *P*4_2_/*mnm*‐(CH_4_)_7_(N_2_)_8_, above 7 GPa and 300 K. Remarkably, compression of either of these compounds results in the breaking of the N_2_ triple bond and dissociation of CH_4_, with the reaction product exhibiting spectroscopic signatures of singly‐bonded C−N−H compounds upon decompression to near‐ambient pressure. We identify the previously predicted molecular compound CH_3_(CH_2_)_2_NH_2_+NH_3_+CH_4_ as a potential reaction product. Upon heating either Ibam ‐(CH_4_)_5_N_2_ or *P*4_2_/*mnm*‐(CH_4_)_7_(N_2_)_8_ to temperatures of 670 K at 14 GPa, we observe decomposition into NH_3_ and longer‐chain light hydrocarbons. Above 1200 K at pressures exceeding 25 GPa, the hydrocarbons further decompose, producing powdered diamond. These combined results demonstrate the complexity of compound formation in the C−N−H ternary system under planetary‐relevant conditions, even when starting from the simplest precursors. Such mixtures comprise the atmosphere of Saturn's moon Titan, and it will be of great interest to see if the reaction products we observe in experiments will be observed directly through the proposed Dragonfly mission exploring prebiotic chemistry on the satellite.

## Conflict of Interests

The authors declare no conflicts of interest.

1

## Supporting information

As a service to our authors and readers, this journal provides supporting information supplied by the authors. Such materials are peer reviewed and may be re‐organized for online delivery, but are not copy‐edited or typeset. Technical support issues arising from supporting information (other than missing files) should be addressed to the authors.

Supporting Information

## Data Availability

The data that support the findings of this study are available from the corresponding author upon reasonable request.
