# Supplementary material for: Pressure‐Driven Reactivity in Dense Methane‐Nitrogen Mixtures
Source: Angew Chem Int Ed Engl. 2025 Apr 3;64(20):e202422710. doi: 10.1002/anie.202422710 (PMC12070456; doi:10.1002/anie.202422710)
Supplement: Supplementary file 1 — Supporting Information [file ANIE-64-e202422710-s001.pdf]

Angewandte Chemie
Eine Zeitschrift der Gesellschaft Deutscher Chemiker

Supporting Information

Pressure-Driven Reactivity in Dense Methane-Nitrogen Mixtures

H. A. Shuttleworth, M. A. Kuzovnikov, L. J. Conway, H. Hu, J. Yan, S. Gallego-Parra, I. Osmond, T. Marqueño, M. Hanfland, D. Laniel, E. Gregoryanz, A. Hermann, M. Peña-Alvarez*, R. T. Howie**

Supporting Information: "Pressure-Driven Reactivity in Dense Methane-Nitrogen Mixtures"

Hannah A. Shuttleworth[1] Mikhail A. Kuzovnikov[1] Lewis J. Conway[3,1] Huixin Hu[4] Samuel Gallego-Parra[2] Israel Osmond [1] Tomas Marqueño[1] Michael Hanfland[2] Dominique Laniel[1] Eugene Gregoryanz[6,5,1] Andreas Hermann*[1] Miriam Peña-Alvarez*[1], Ross T. Howie*[1,4]

[1]Centre for Science at Extreme Conditions, the University of Edinburgh, Edinburgh, EH9 3FD, U.K.
[2]European Synchrotron Radiation Facility, 71 Avenue des Martyrs, 38000 Grenoble, France
[3]Department of Materials Science & Metallurgy, University of Cambridge, 27 Charles Babbage Road, Cambridge CB3 0FS, United Kingdom
[4]Center for High Pressure Science and Technology Advanced Research, 1690 Cailun Road, Shanghai, 201203, China
[5]Key Laboratory of Materials Physics, Institute of Solid State Physics, CAS, Hefei, 230031, China
[6]SHARPS (Shanghai Advanced Research in Physical Sciences), 68 Huatuo Road, Shanghai, 201203, China
*ross.howie@ed.ac.uk, miriam.pena.alvarez@ed.ac.uk, a.hermann@ed.ac.uk

Experimental Methods and Analysis

Sample Preparation

Experiments were primarily performed on two representative gas mixtures of fixed concentrations: BOC 50% Methane/Nitrogen Research Grade (99.999%) and 67% Methane/33% Nitrogen Research Grade (99.999%). Additional experiments were also performed on 20% Methane/80% Nitrogen Research Grade (99.999%) and 90% Methane/10% Nitrogen Research Grade mixtures. All gas mixtures had a gas tolerance of ±1%. A small grain of Au was loaded into the sample chambers to determine the pressure inside the sample chamber in X-ray diffraction measurements [1], along with a ruby sphere for Raman spectroscopy experiments (where the pressure did not exceed 50 GPa) [2]. For Raman spectroscopy experiments exceeding 50 GPa, the stressed Raman edge of the diamond anvil was used to determine pressure [3]. Pressures were generated using symmetric diamond-anvil cells (DACs) of our design equipped with diamonds that have culet diameters ranging from 70 µm to 200 µm. Rhenium foils 200-250 µm thick were used as a gasket material. The gas mixtures were loaded using an in-house gas loading system at pressures of ∼2000 bars.

X-ray Diffraction Measurements

The single crystal X-ray diffraction studies were performed at ID15-b beamline at the ESRF, Grenoble, France (measurements included in manuscript Figs. 1c and 2b) [4] and the Extreme Conditions Beamline (ECB, P02.2) at PETRA-III, Hamburg, Germany (measurements included in Figs. 1b and 2b) [5]. A full X-ray diffraction mapping of the sample chamber is performed to determine the sample position where the optimum single crystal X-ray diffraction acquisition is obtained. The sample position displaying the most single crystal reflections belonging to the phase of interest is chosen for the collection, in step-scans of 0.5° across the whole aperture of the DAC. The CrysAlis^{Pro} software is utilized for the single crystal data analysis [6]. The analysis procedure includes a peak search, the removal of the diamond anvils' and other 'parasitic' signal contributions, finding reflections belonging to a unique single crystal, the unit

cell determination, and the data integration. The crystal structures were then solved and refined using the OLEX2 software [7]. For σCrFe-$(CH_4)_7(N_2)_8$, the atoms' thermal parameters were anisotropically refined, providing qualitative information on the preferred orientation of the rotationally disordered molecules, as demonstrated in Fig. 1a.

When refining the *Ibam* compound, significant electronic density was found on six distinct crystallographic positions, five on the $8j$ Wyckoff site, and one on the $16k$ Wyckoff site. Nitrogen could unambiguously be assigned to the $16k$ site as it produces an ordered dimer with a bond length of 0.993(4) Å, matching what is expected of molecular N_2 [8]. The five $8j$ sites were assigned to carbon atoms, representing CH_4 molecules. The presence of a rotationally disordered N_2 molecule on any of the $8j$ sites could be ruled out as these represent substantially more electronic density than a CH_4 molecule, significantly more than suggested by experimental data. When nonetheless attempted by replacing a C atom with a Si atom (with 14 electrons, the same as an N_2 molecule), it resulted in unreasonable thermal parameters (for example 0.203 Å^2).

Angular-dispersive powder X-ray diffraction experiments were performed at the ID15-b beamline at the ESRF, Grenoble, France, (measurements included in manuscript Figs. 2b and 4b, supplementary Figs. S1a and S4) [4], the Extreme Conditions Beamline (ECB, P02.2) at PETRA-III, Hamburg, Germany (measurements included in manuscript Figs. 2b, and Supplementary Fig. S5) [5], at the I15 beamline at Diamond Light Source, Didcot, UK (measurements included in Figs. S1b and manuscript Fig. 2b) [9], and at the BL10XU beamline at SPring-8, Hyogo, Japan (measurements included in Fig. 2b) [10].

Typically, the cell was oscillated around the ω axis by $\pm 15°$, and the 2D diffraction image was collected for 20-30 s. At P02.2, we used an incident X-ray beam with E = 42.4 keV or 25.5 keV ($\lambda \sim$ 0.292 Å or $\sim$ 0.4841 Å). Diffraction images were recorded using Perkin Elmer XRD1621 detector with a sample-to-detector distance (SDD) of 400 mm, as calibrated with a CeO_2 standard. At ID15-b an incident beam with E $\sim$ 30.2 keV ($\lambda \sim$ 0.410 Å) was used. The diffraction patterns were collected with EIGER2 X 9M detector with SDD $\sim$ 181 or 250 mm, calibrated using a Si powder standard. At I15 an incident beam had E $\sim$ 29.2 keV ($\lambda \sim$ 0.425 Å). We used Pilatus3 2M detector with SDD $\sim$ 403 mm, calibrated with a LaB_6 standard. At BL10XU we used an incident beam with E $\sim$ 30.0 keV ($\lambda \sim$ 0.414 Å). The diffraction patterns were collected with Perkin Elmer XRD0822 detector with SDD $\sim$ 300 mm, calibrated using a CeO_2 powder standard. The SDD, detector orientation and wavelength calibration, primary processing, azimuthal integration, and background subtraction were performed with the DIOPTAS v0.5.5 software [11]. Phase analysis and Rietveld refinements were performed with the POWDERCELL 2.4 program [12].

Raman Spectroscopy Measurements

Raman spectroscopy measurements were conducted using a custom-built confocal setup in 180deg backscattering geometry. A 514.5 nm Ar-ion laser (Coherent Innova 70C) and a 647.1 nm Kr-ion (Coherent Innova 300C) were used as the excitation sources. An Acton SP-2500i spectrometer (Princeton Instruments) equipped with a with 1800 gr/mm, 1200 gr/mm and 300 gr/mm gratings coupled with PyLoN CCD (Princeton Instruments) were used.

Resistive and Laser Heating Techniques

Resistive-heated high-temperature experiments were conducted using modified high-temperature DACs equipped with a primary and a secondary heater, and thermocouples. A type-K thermocouple was partially clamped between the gasket and the diamond anvil. Good mechanical contact ensures a more accurate temperature measurement while ensuring proximity to the sample chamber. Heating was done in two stages: (i) the primary, external to the cell assembly heated to a maximum of 500 °C; and (ii) a secondary internal heater, situated around the diamond anvils, heating to a maximum of 730 °C.

In separate experiments, samples were laser heated *in-situ* by directly coupling to a yttrium-aluminum-garnet (YAG) continuous wave lasers with wavelength λ=1064 nm. We estimate temperatures of 1200 $\pm$ 50 K from the detected black-body radiation.

Density Functional Calculations

Computational Methods

Density functional theory calculations were carried out using the CASTEP software package [13]. For the σ-CrFe structure, with 30 molecules per unit cell, the calculation of 2^{30} site occupancies (which still disregards molecular orientations) was not feasible. Therefore, we constructed ordered variants of the σ-CrFe structure where the individual Wyckoff sites are fully occupied with either N_2 or CH_4. This resulted in $2^5 = 32$ variants across 16 different compositions. For each variant, several initial structures were constructed with random molecular orientations and subsequently optimized fully. The same computational method was applied to estimate the composition of the *Ibam* structure. This structure has six distinct crystallographic sites, which we occupied fully with either N_2 or CH_4. The resulting 64 variants take up one of seven different compositions. For each such variant, several initial structures were constructed with random molecular orientations, before full structure optimization at 15 GPa. Ultrasoft pseudopotentials modeled the electron-ion interactions, the Perdew-Burke-Ernzerhof functional modeled the electronic exchange-correlation interactions [14], and a plane wave basis set with kinetic energy cutoff $E_c = 1000$ eV was used to expand the electronic wavefunctions. All structures were optimized until the residual forces on the atoms were below 50 meV/Å.

Molecular dynamics (MD) simulations were performed for both structures at 15 GPa and 300 K, using (1,1,2) supercells of the previously optimized crystal structures; these cells contained 204 (432) atoms for the σ-CrFe (*Ibam*) structures. For the MD calculations, the plane wave cutoff was reduced to $E_c = 600$ eV.

The role of dispersion interactions was explored by re-optimizing the best compounds from each composition and structure type with two different dispersion-corrected exchange-correlation functionals, PBE+TS [15] and PBE+MBDrsSCS (Supporting Fig. S8) [16].

Molecular Dynamics Results

The main results from the MD simulations are summarised in Figure S6. The atomic mean square displacements (MSD's) are consistent with rotational CH_4 molecules, while N_2 molecules experience librations around their equilibrium positions. For the *Ibam* phase this is consistent with the single crystal diffraction results. The trajectories shown are in agreement with the MSD data: methane's hydrogen atoms move freely on spherical surfaces around the central carbon atoms, while the latter (as well as nitrogen) remain close to their crystalline sites.

Role of Dispersion Corrections

The main manuscript summarises the energetics of the different phases in a convex hull plot of formation enthalpies. Figure S7 shows the corresponding volumes of these phases, all obtained at 15 GPa, together with the experimental volumes of pure nitrogen, methane, and the two new phases, also at 15 GPa. The DFT volumes for the end members agree well with experiment, and all mixtures exhibit essentially a linear dependence on composition. The DFT volumes and enthalpies depend on the choice of exchange-correlation functional. Even though both new phases form at high pressure, the lack of bonding between their constituent molecules suggests that weak dispersion interactions might play a role in their formation. Figure S8 shows both energetics and volume data from two dispersion-corrected functionals. For both functionals, at 15 GPa, no compounds are on the convex hull; however, the two phases proposed here are closest to stability in both cases. All volumes are lower, and for pure nitrogen and methane are significantly smaller than seen in experiment. This is most pronounced for methane, so we conjecture that both functionals over-stabilize pure methane, and therefore underestimate compound stabilities.

Supporting Tables and Figures

Pressure (GPa)		7	
Space group, #		$P4_2/mnm$, 136	
a (Å)		11.904(3)	
b (Å)		11.904(3)	
c (Å)		6.2072(13)	
V (Å^3)		879.6(5)	
**Refinement details**			
Wavelength (λ, Å)		0.2899	
μ (mm^{-1})		0.097	
# measured reflections (I $\geq 2\sigma$)		1179 / 309 (136)	
$(\sin\theta/\lambda)_{\max}$ (Å^{-1})		0.627	
R_{int} (%)		9.71	
$R[F^2/\sigma(F^2) > 2]$ (%)		14.69	
wR(F2) (%)		41.98	
S		1.239	
No. of parameters		26	
$\Delta\rho$min, $\Delta\rho$max (eÅ^{-3})		-0.53, 0.63	
**Atomic positions**			
Atom	Wyckoff position	Fractional atomic coordinates (x; y; z)	U_{iso} (Å^2)
Si01	$2a$	1/2 1/2 1/2	0.156(5)
Si02	$8i$	0.5630(6) 0.7626(5) 1/2	0.132(3)
Si03	$4f$	0.1005(5) 0.8995(5) 1/2	0.114(3)
Si04	$8i$	0.3703(6) 0.9610(5) 1/2	0.131(3)
Si05	$8j$	0.8159(5) 0.8159(5) 0.7464(5)	0.162(4)

Table S1: Crystallographic data for a σCrFe-$(CH_4)_7(N_2)_8$ single-crystal at 7 GPa. The refinement was done with the OLEX2 software [7]. To model the rotational and site disorder of the N_2 and CH_4 molecules, they were both represented by Si atoms. The atoms' thermal parameters were anisotropically refined, providing some clue on the preferred orientation of the rotationally disordered molecules.

Pressure (GPa)		13	
Space group, #		*Ibam*, 72	
a (Å)		11.854(12)	
b (Å)		18.459(5)	
c (Å)		5.6278(12)	
V (Å^3)		1231.5(13)	
**Refinement details**			
Wavelength (λ, Å)		0.4099	
μ (mm^{-1})		0.026	
# measured reflections (I $\geq 2\sigma$)		946/438 (243)	
$(\sin\theta/\lambda)_{\max}$ (Å^{-1})		0.609	
R_{int} (%)		0.224	
R[F^2/σ(F^2) > 2] (%)		9.34	
wR(F2) (%)		28.70	
S		1.030	
No. of parameters		40	
$\Delta\rho$min, $\Delta\rho$max (eÅ^{-3})		-0.15, 0.17	
**Atomic positions**			
Atom	Wyckoff position	Fractional atomic coordinates (x; y; z)	U_{iso} (Å^2)
C1	8j	0.2066(2) 0.2236(5) 0	0.0727(18)
C2	8j	0.0588(2) 0.4094(5) 0	0.0772(18)
C3	8j	0.1046(2) 0.3064(6) 1/2	0.082(2)
C4	8j	0.1535(2) 0.0637(4) 1/2	0.0695(18)
C5	8j	0.0397(2) 0.1284(6) 0	0.092(2)
N1	16k	0.20247(15) 0.4571(3) 0.2490(6)	0.087(3)

Table S2: Crystallographic data for *Ibam*-$(CH_4)_5N_2$ single-crystal at 13 GPa. The refinement was done with the Olex2 software [7].

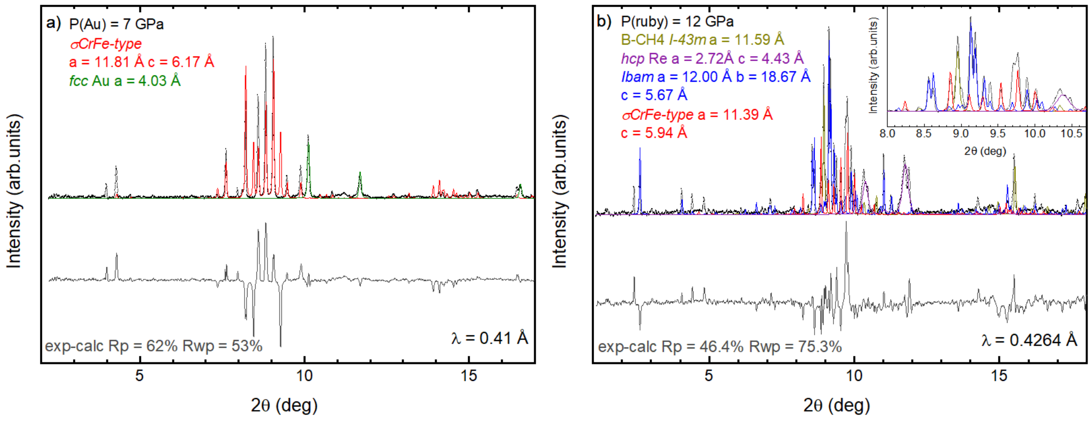

Figure S1: Rietveld refinements of (a) σCrFe-$(CH_4)_7(N_2)_8$ and gold at 7 GPa and room temperature (b) *Ibam*-$(CH_4)_5N_2$ along with σCrFe-$(CH_4)_7(N_2)_8$, *hcp* Re and B-CH_4 at 12 GPa and room temperature. The upper right corner provides a zoomed panel so that the peaks around 2θ=8.0-10.7^o may be resolved. *Ibam*-$(CH_4)_5N_2$ was never observed in a pure phase, suggesting that the reaction B-CH_4+σCrFe-$(CH_4)_7(N_2)_8$ $\rightarrow$ *Ibam*-$(CH_4)_5N_2$ is slow and does not undergo completion.

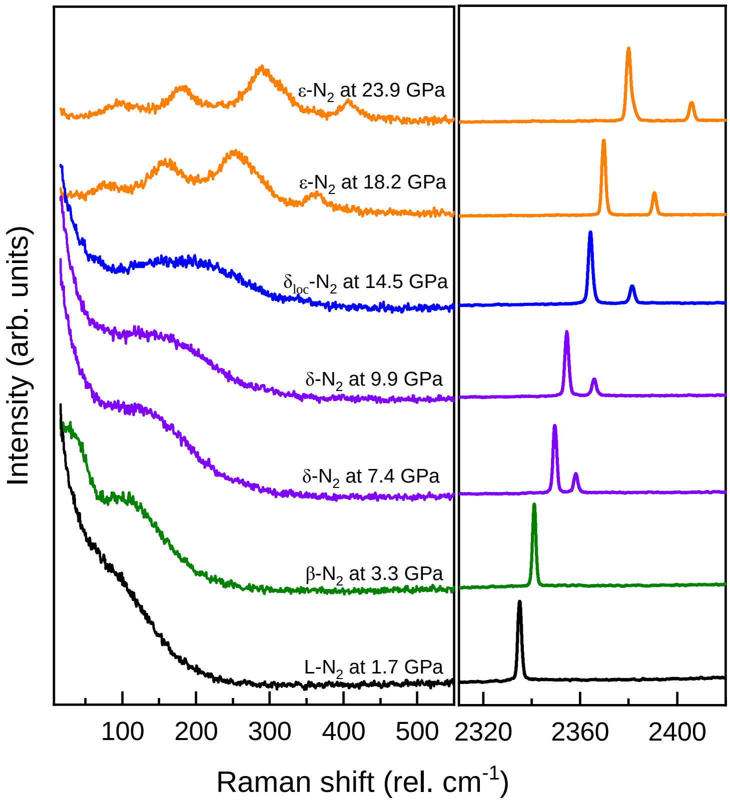

Figure S2: Raman spectra as a function of pressure of pure N_2 at 300 K up to 23.9 GPa. Different colors represent different phases of N_2.

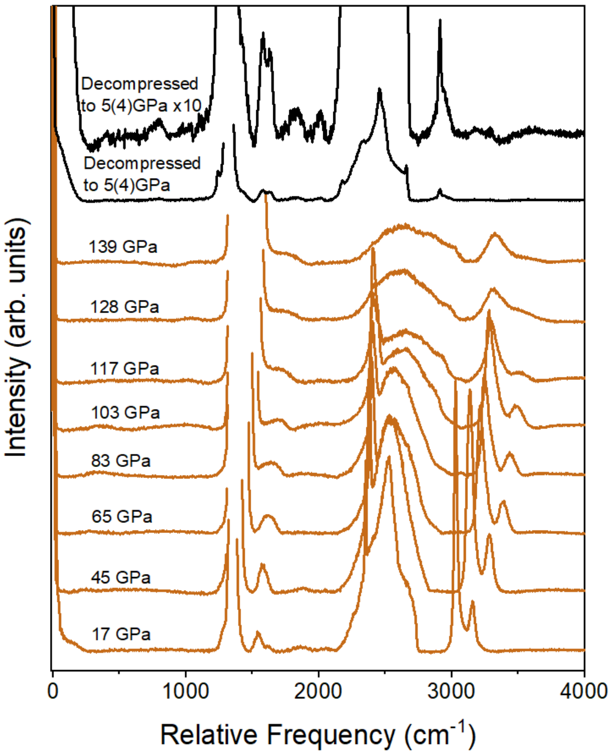

Figure S3: Raman spectra upon compression of *Ibam*-$(CH_4)_5N_2$, synthesized from a 67% CH_5 mixture, from 17 to 139 GPa (orange) at room temperature, where the softening and ultimately the disappearance of the N_2 vibrational mode can be seen, and Raman spectra after decompressing the same sample to 5(4) GPa (black).

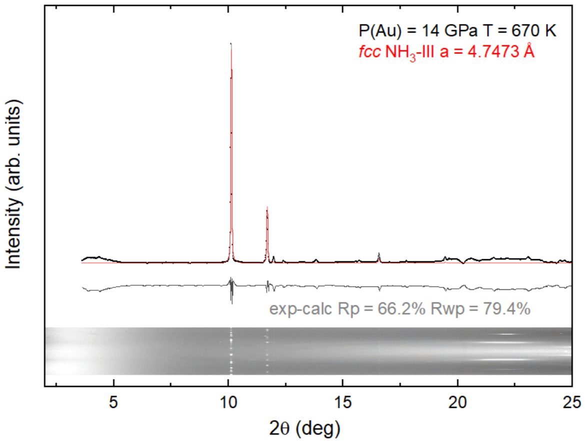

Figure S4: Rietveld refinement of *fcc* NH_3-III at 670 K and 14 GPa, formed by resistively heating *Ibam*-$(CH_4)_5N_2$.

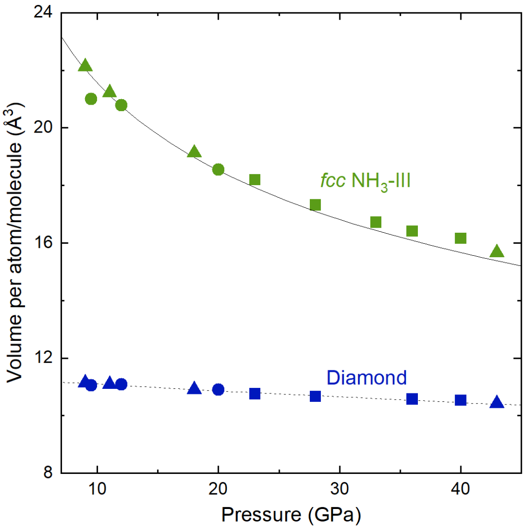

Figure S5: Volume as a function of pressure, calculated from powder XRD refinements, upon decompression after laser heating $Ibam$-$(CH_4)_5N_2$ at 43 GPa and 1200 K to form NH_3-III, hydrocarbons and powdered diamond. Different symbols represent different experimental runs. The solid line gives the previously-established equation of state of NH_3 [17], and the dotted line represents the equation of state of diamond [18].

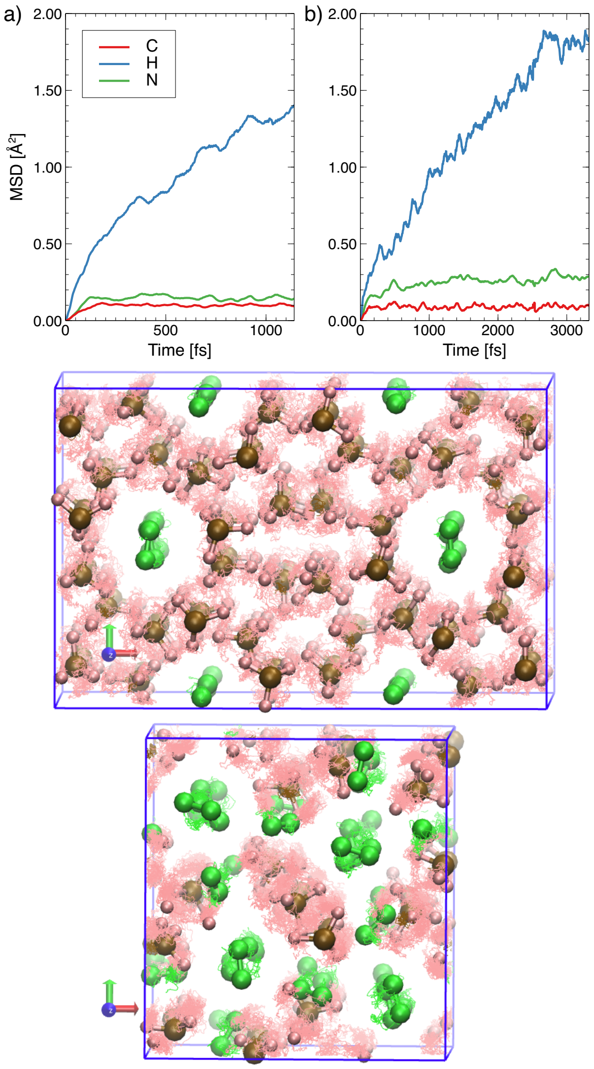

Figure S6: Top: atomic mean square displacement (MSD) from MD calculations for the a) *Ibam* and b) σ-CrFe structure. Middle: the full trajectory of the *Ibam* structure. Bottom: the full trajectory of the σ-CrFe structure. Brown/green/pink spheres and points denote C/N/H atoms, respectively.

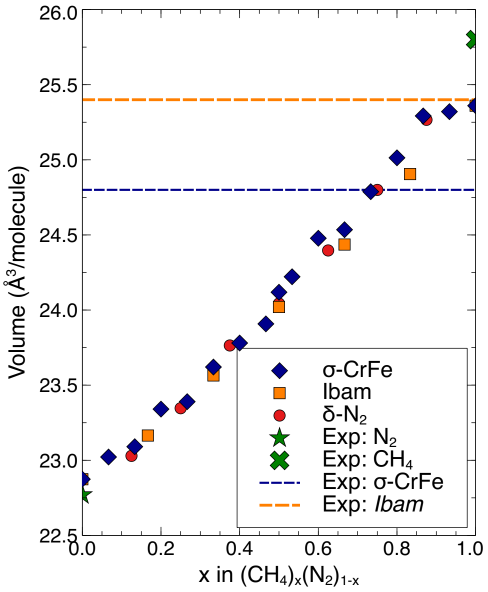

Figure S7: Volume as a function of composition from DFT PBE calculations at 15 GPa, displaying the volume per molecule for the enthalpically most stable structures constructed from the different structure types (see legend); i.e., the same structures shown in Fig. 2a of the main manuscript. Experimental data has been extracted from Fig. 2b of the main manuscript.

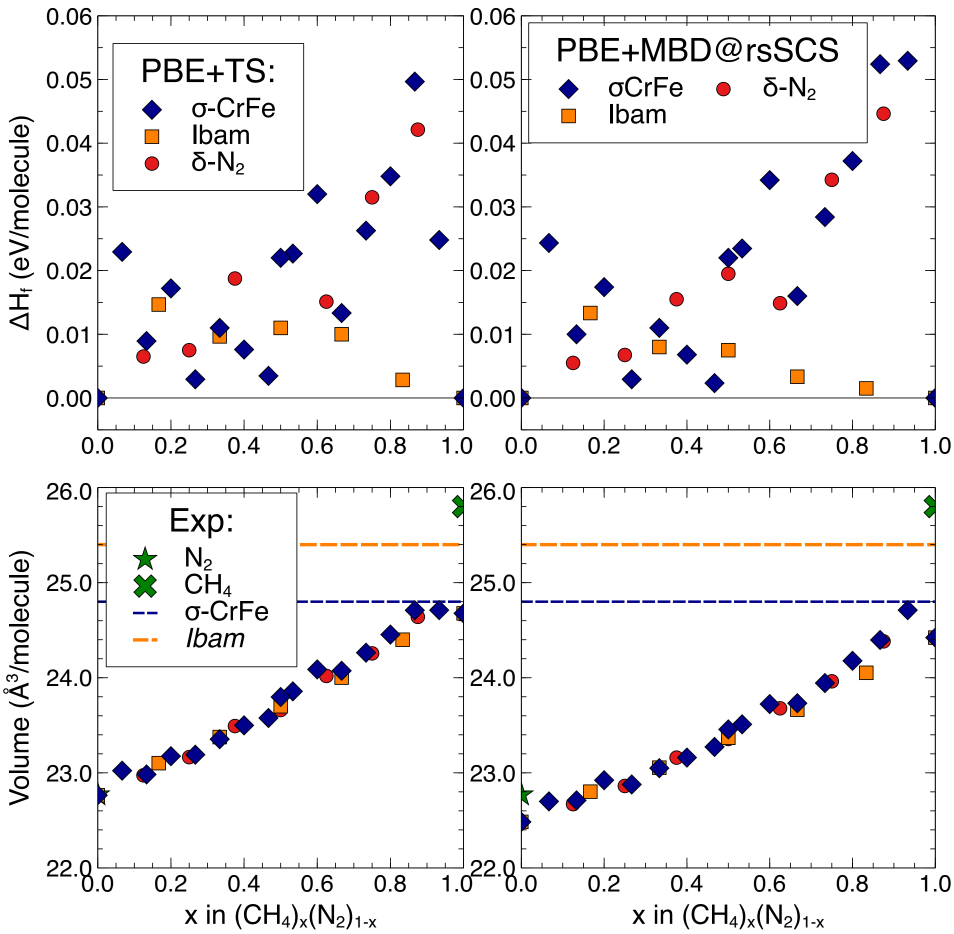

Figure S8: Top row: convex hull diagrams at 15 GPa, obtained from PBE+TS (left) and PBE+MBDrsSCS (right) calculations. Bottom row: corresponding volumes per molecule for the same phases; experimental data (see Fig. S7 included as reference).

References

[1] S. M. Dorfman, V. B. Prakapenka, Y. Meng, and T. S. Duffy, Journal of Geophysical Research: Solid Earth **117**, B08210 (2012).

[2] H. Mao, J.-A. Xu, and P. Bell, Journal of Geophysical Research: Solid Earth **91**, 4673 (1986).

[3] Y. Akahama and H. Kawamura, Journal of Applied Physics **100**, 043516 (2006).

[4] M. Merlini and M. Hanfland, High Pressure Research **33**, 511 (2013).

[5] H.-P. Liermann, Z. Konôpková, W. Morgenroth, K. Glazyrin, J. Bednarčik, E. McBride, S. Petitgirard, J. Delitz, M. Wendt, Y. Bican, et al., Journal of synchrotron radiation **22**, 908 (2015).

[6] *Rigaku Oxford Diffraction. CrysAlisPro Software system.* (2015).

[7] O. V. Dolomanov, L. J. Bourhis, R. J. Gildea, J. A. Howard, and H. Puschmann, Journal of applied crystallography **42**, 339 (2009).

[8] M. Hanfland, M. Lorenzen, C. Wassilew-Reul, and F. Zontone, The Review of High Pressure Science and Technology **7**, 787 (1998).

[9] S. Anzellini, A. K. Kleppe, D. Daisenberger, M. T. Wharmby, R. Giampaoli, S. Boccato, M. A. Baron, F. Miozzi, D. S. Keeble, A. Ross, et al., Journal of synchrotron radiation **25**, 1860 (2018).

[10] Y. Ohishi, N. Hirao, N. Sata, K. Hirose, and M. Takata, High pressure research **28**, 163 (2008).

[11] C. Prescher and V. B. Prakapenka, High Pressure Research **35**, 223 (2015).

[12] W. Kraus and G. Nolze, Journal of Applied Crystallography **29**, 301 (1996).

[13] S. J. Clark, M. D. Segall, C. J. Pickard, P. J. Hasnip, M. I. J. Probert, K. Refson, and M. C. Payne, Z. Kristall. **220**, 567 (2005).

[14] J. P. Perdew, K. Burke, and M. Ernzerhof, Phys. Rev. Lett. **77**, 3865 (1996).

[15] A. Tkatchenko and M. Scheffler, Phys. Rev. Lett. **102**, 073005 (2009).

[16] A. Ambrosetti, A. M. Reilly, R. A. DiStasio, and A. Tkatchenko, The Journal of Chemical Physics **140**, 18A508 (2014).

[17] F. Datchi, S. Ninet, M. Gauthier, A. Saitta, B. Canny, and F. Decremps, Physical Review B **73**, 174111 (2006).

[18] F. Occelli, P. Loubeyre, and R. LeToullec, Nature materials **2**, 151 (2003).
